# A CRISPR-Cas12a-Assisted Fluorescence Platform for Rapid and Accurate Detection of *Nocardia cyriacigeorgica*

**DOI:** 10.3389/fcimb.2022.835213

**Published:** 2022-03-02

**Authors:** Xueping Liu, Xiaotong Qiu, Shuai Xu, Yanlin Che, Lichao Han, Yutong Kang, Yuan Yue, Shenglin Chen, Fang Li, Zhenjun Li

**Affiliations:** ^1^School of Laboratory Medicine and Life Sciences, Wenzhou Medical University, Wenzhou, China; ^2^State Key Laboratory of Infectious Disease Prevention and Control, National Institute for Communicable Disease Control and Prevention, Chinese Center for Disease Control and Prevention, Beijing, China; ^3^Key Laboratory of the Ministry of Education for the Conservation and Utilization of Special Biological Resources of Western China, Ningxia University, Yinchuan, China; ^4^School of Public Health, Shanxi Medical University, Taiyuan, China; ^5^Department of Medicine, Tibet University, Lhasa, China

**Keywords:** *Nocardia cyriacigeorgica*, nucleic acid detection, CRISPR, Cas12a, CRISPR-PCR

## Abstract

*Nocardia cyriacigeorgica* has gradually become a common pathogen in clinical microbial infections. Identification of *Nocardia* at the species level is essential to assess the susceptibility and pathogenicity of antimicrobials. However, there is no suitable method for rapid and accurate laboratory detection of *N. cyriacigeorgica*. In this study, we combined PCR amplification with the CRISPR-Cas12a system to establish a novel detection platform, named CRISPR-PCR, and applied it to the detection of *N. cyriacigeorgica* in clinical samples. The Cas12a protein exhibited collateral cleavage activity following CRISPR RNA binding to specific targets, then indiscriminately cleaved nearby single-stranded DNA, and this was evaluated for diagnostic nucleic acid detection by measuring the fluorescence signal using a fluorescence reader. The assay takes only 2 h, including DNA extraction for 20 min, nucleic acid pre-amplification for 70 min, and fluorescence detection for 20 min. The limit of detection for *N. cyriacigeorgica* was 10^-3^ ng and the specificity was 100%. Thus, the *N. cyriacigeorgica* CRISPR-PCR assay is a rapid and specific method for detecting *N. cyriacigeorgica*, and the CRISPR-PCR fluorescence detection platform has great potential for detection of other pathogens.

## Introduction

*Nocardia* bacteria are Gram-positive and partially acid-fast aerobic *Actinomycetes* (McTaggart et al., 2010). The genus *Nocardia* is ubiquitous in environments associated with decaying vegetation and deposits from animals, and members are also associated with water, soil and air (Brown-Elliott et al., 2006; Lebeaux et al., 2019). *Nocardia* can cause serious opportunistic infection in most organs, including traumatic infection, pulmonary disease and brain abscess (McTaggart et al., 2010; Huang et al., 2019; Lebeaux et al., 2019; Ji et al., 2020). The incidence of *Nocardia* infection is increased significantly in immunocompromised individuals (Minero et al., 2009; Coussement et al., 2016), including advanced acquired immunodeficiency syndrome (AIDS) (Castro and Espinoza, 2007), patients undergoing organ transplantation (Coussement et al., 2016) and chronic lung disease.

More than 100 species belonging to the genus *Nocardia* have been recognised according to the National Center for Biotechnology Information (NCBI), and more than 50 species are of clinical significance (Conville et al., 2018). *Nocardia cyriacigeorgica* is one of the most common agents of *Nocardia* infection. With an increase in immunocompromised patients and improvements in molecular identification of pathogens, the incidence of *N. cyriacigeorgica* infection is rising (Condas et al., 2013; Hashemi-Shahraki et al., 2015; Chen et al., 2017; Han et al., 2020).

Identification of *Nocardia* at the species level is essential to assess the susceptibility and pathogenicity of antimicrobials. Different *Nocardia* species have different antimicrobial susceptibility patterns, which may cause confusion during clinical treatment (Huang et al., 2019; Hamdi et al., 2020). The genome sequences of *Nocardia* species are highly similar (Conville et al., 2018), hence biochemical identification methods are not adequate to allow discrimination among species. The gold standard for *N. cyriacigeorgica* identification is 16S rDNA sequencing, but this is time-consuming and it requires sophisticated equipment, which makes it challenging for routine laboratories (Barnaud et al., 2005; McTaggart et al., 2010). Thus, there is a growing need for a detection platform that can rapidly and accurately identify *N. cyriacigeorgica*.

The clustered regularly interspaced short palindromic repeats and CRISPR-associated protein (CRISPR-Cas) system is an adaptive immune system in bacteria and archaea that recognises and degrades foreign nucleic acid through the guidance of CRISPR RNA (crRNA) (Abudayyeh et al., 2016; Chen et al., 2018; Xiong et al., 2020). Cas proteins, including cas12a, cas12b, cas13a, cas13b and cas14, recognize and *cis-*cleavage target DNA or RNA, and then activated Cas proteins *trans-*cleavage non-targeted single-stranded DNA (ssDNA) indiscriminately (Gootenberg et al., 2017; Harrington et al., 2018; Li et al., 2018; Wang et al., 2019). Therefore, non-targeted ssDNA labelled with a fluorophore and quencher produces a fluorescence signal after collateral cleavage, which can be used to detect nucleic acids (Huang et al., 2020; Xiong et al., 2020). The intensity of fluorescence signals can be measured using a fluorescence reader for specific nucleic acid detection (Zhang et al., 2021).

Recently, detection methods based on the CRISPR-Cas system have been developed, and they show great promise. The CRISPR-Cas system is commonly combined with isothermal amplification or PCR amplification to rapidly and accurately detect nucleic acids for pathogen identification (Kellner et al., 2019). Isothermal amplification, such as recombinase polymerase amplification (RPA) and loop-mediated isothermal amplification (LAMP), can be combined with Cas12 or Cas13 for nucleic acid detection. Specific high-sensitivity enzymatic reporter (SHERLOCK) can be combined with CRISPR-Cas13a and RPA to specifically detect Zika and Dengue viruses (Gootenberg et al., 2017; Kellner et al., 2019). CRISPR-Cas12a-based detection can rapidly and accurately distinguish human papillomavirus (HPV) type 16 and type 18 using the DNA endonuclease-targeted CRISPR trans reporter (DETECTR) (Chen et al., 2018) and 1-hour low-cost multipurpose highly efficient system (HOLMES) approach (Li et al., 2018). However, isothermal amplification suffers limitations; it is prone to false-positive results and it is expensive.

**Figure 1:**
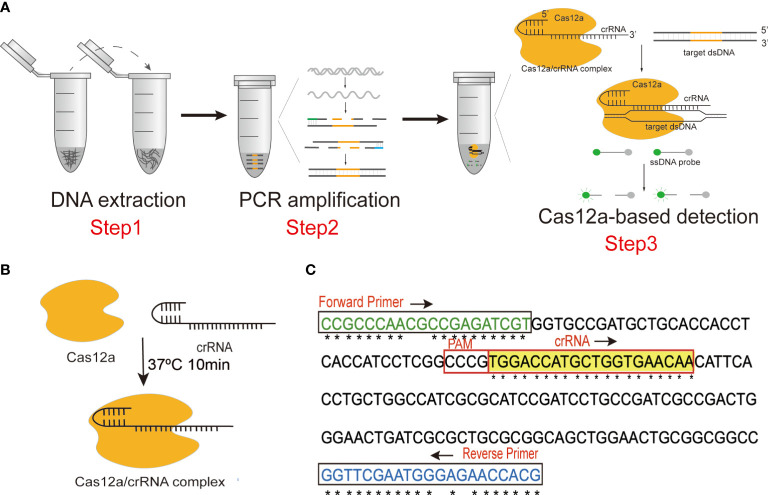
**(A)** Schematic diagram of the *N. cyriacigeorgica* CRISPR-PCR assay. **(B)** Schematic diagram of the CRISPR-Cas12a/crRNA complex. **(C)** Sequences and locations of PCR primers and crRNA in this assay.

In the present study, we established a highly accurate and sensitive detection platform combining PCR amplification with CRISPR-Cas12a, termed CRISPR-PCR, to detect *N. cyriacigeorgica* (**Figure 1A**). Target sequences were amplified by PCR, then CRISPR-Cas12a was combined with crRNA to generate a CRISPR-Cas12a/crRNA complex (**Figure 1B**), which exhibited collateral cleavage of a ssDNA probe to generate a fluorescence signal. The *N. cyriacigeorgica* CRISPR-PCR assay can directly detect low levels of *N. cyriacigeorgica* in clinical samples, and it could help clinicians to employ appropriate antibiotic regimes in the early stages of infection.

## Materials and Methods

### Materials

Primers for PCR amplification, ssDNA probe, and crRNA were synthesised by Sangon Biotech (Shanghai, China) with high-performance liquid chromatography (HPLC) purification (**Table S1**). LbaCas12a and 10× NEBuffer 2.1 were purchased from New England Biolabs (Beijing, China). A Wizard Genomic DNA Purification Kit was purchased from Promega (USA). DNase/RNase-free deionised water was purchased from TIANGEN (Beijing, China). Premix Ex Taq (Probe qPCR) was purchased from TaKaRa (Dalian, China). A QuantStudio™ 6 Flix instrument (X) was employed as a fluorescence reader.

### Oligonucleotide Sequence Design

The *Ncc1* gene of *N. cyriacigeorgica* (Accession: LR215973.1), which encodes an uncharacterised protein (Protein ID: VFA97995.1), was used for primer design. The specificity of *Ncc1* was verified by BLASTn. Clustal Omega (https://www.ebi.ac.uk/Tools/msa/clustalo/) was employed for multiple sequence alignment to identify conserved regions of *Ncc1*, which served as target sequences (**Figure 1C** and **Figure S1**).

PCR primer pairs targeting conserved sequences of *Ncc1* were designed using standard procedures (**Figure 1C** and **Table S1**). We screened the best primer pair and the optimum annealing temperature for subsequent experiments. The specificity of the primer pair was verified by Primer-BLAST (https://www.ncbi.nlm.nih.gov/tools/primer-blast/).

Non-targeting ssDNA fluorescent probes can be constituted by thymine polymer (poly T). The ssDNA was modified by adding the fluorescent group 6-FAM at the 5’ end and the quencher group BHQ1 at the 3’ end (**Table S1**). To achieve high detection specificity, we used the highly-conserved region to design an efficient crRNA according to the CRISPR-Cas12a detection principle (**Figure 1C** and **Table S1**).

### Establishing the CRISPR-PCR Fluorescence Detection Assay

A DNA fragment of the *Ncc1* gene was selected as the target sequence (**Figure 1C**), which was amplified by PCR in a 20 μL reaction containing 10 μL of Premix Ex Taq (Probe qPCR), 7 μL of DNase/RNase-free deionised water, 1 μL of forward primer (10 μM), 1 μL of reverse primer (10 μM), and 1 μL of template DNA. Thermal cycling involved an initial denaturation for 5 min at 95°C followed by 35 cycles at 95°C for 30 s, 65°C for 30 s, 72°C for 30 s, and a final extension for 7 min at 72°C.

The Cas12a protein was combined with crRNA to form a CRISPR-Cas12a/crRNA complex (**Figure 1B**) in advance to stabilise the CRISPR detection process. CRISPR-Cas12a/crRNA complexes contained 100 nM crRNA and 75 nM Cas12a in 1× NEBuffer 2.1, they were incubated at 37°C for 10 min, and they were used immediately after preparation. The 100 μL reaction volume of the CRISPR-Cas12a system included 50 μL of 2× NEBuffer 2.1, 18 μL of CRISPR-Cas12a/crRNA complex, 2 μL of amplification product, 27.5 μL of distilled water, and 2.5 μL of 10 μM ssDNA probe. Fluorescence signals were detected by a fluorescence reader at 37°C for 20 min. Fluorescence measurements were performed every minute to determine whether the sample contained the target sequence.

### Assessing the Specificity and Sensitivity of the *N. cyriacigeorgica* CRISPR-PCR Assay

The specificity of the *N. cyriacigeorgica* CRISPR-PCR assay was verified by 60 N. *cyriacigeorgica* strains (including 7 standard strains and 53 clinical strains) and 44 non-*N. cyriacigeorgica* strains (including 25 type strains of other *Nocardia* species and 19 strains not from the *Nocardia* genus; **Table S2**).

The genome of the *N. cyriacigeorgica* type strain (DSM 44484) was extracted using a Wizard Genomic DNA Purification Kit. The initial DNA concentration was 100 ng/μL measured by a NanoDrop-1000 instrument. To define the limit of detection (LoD) of the *N. cyriacigeorgica* CRISPR-PCR assay, we serially diluted template DNA 10-fold intervals from 10 ng to 10^-4^ ng, and 1 μL aliquots of templates were used in CRISPR-PCR reactions. Agarose gel analysis was a control experiment for sensitivity detection.

### Applying the *N. cyriacigeorgica* CRISPR-PCR Assay to Clinical Samples

To evaluate the practical applicability of the *N. cyriacigeorgica* CRISPR-PCR assay, we assessed clinical samples. Since *N. cyriacigeorgica* is difficult to identify in routine laboratories, and because *Nocardia* infection is relatively rare, we spiked *N. cyriacigeorgica* bacteria liquid into clinical samples to mimic clinical situations. To assess assay sensitivity, 900 μL aliquots of sputum specimens were inoculated with 100 μL of a calibrated suspension of *N. cyriacigerogica* at a final concentration ranging from 10^6^ colony-forming units (CFU)/mL to 10^2^ CFU/mL. The amplified products were separated by 2% agarose gel electrophoresis. Isolated cultures were used in a parallel experiment to assess the specificity of clinical sample detection. Briefly, 20 negative sputum specimens, which were shown to be free of *N. cyriacigerogica* by isolated culture, were divided into two equal parts; one part was used for preparation of positive sputum specimens, and the other served as a negative control. The 20 positive sputum specimens consisted of 900 μL of sputum and 100 μL of bacterial liquid, and equal volumes were added to 100 μL of phosphate-buffered saline as negative controls. Nucleic acids extracted from the 40 sputum specimens were used as amplification templates. For this, 1 μL of DNA template was added to the PCR system for amplification, and 2 μL of the resulting amplicons were added to the CRISPR reaction system. Fluorescence signals were detected by a fluorescence reader at 37°C for 20 min.

## Results

### Establishing the *N. cyriacigeorgica* CRISPR-PCR Assay

We established the *N. cyriacigeorgica* CRISPR-PCR assay by combining the CRISPR-Cas12a system and PCR amplification to identify *N. cyriacigeorgica* DNA accurately and robustly, and the assay can be performed in routine laboratories. Our CRISPR-PCR assay can achieve rapid detection, and can be completed within 2 h. The assay employs three steps, including DNA extraction, target amplification, and fluorescence signal detection (**Figure 1A**). We amplified part of the *Ncc1* gene by PCR and added amplicons to the CRISPR-Cas12a reaction system. The CRISPR-Cas12a/crRNA complex specifically binds and cleaves target dsDNA (*cis-*cleavage), which is complementary to crRNA, then activates collateral cleavage activity which degrades ssDNA probe (*trans-*cleavage) indiscriminately to produce fluorescence. Fluorescence signals can be observed by a fluorescence reader. Notably, the assay can potentially be used for point-of-care testing if results are analysed with a homemade UV device, by the naked eye, or by a portable fluorescence reader.

### Optimising the *N. cyriacigeorgica* CRISPR-PCR Assay

Seven forward primers and five reverse primers were designed targeting the conserved region of the *Ncc1* gene (**Figure 1C**, **Table S1** and **Figure S1**). The mutation sites were replaced with degenerate bases (N). In order to screen the best primer pair, a reverse primer R4 was used with forward primers F1 to F7 (**Figure S2A**). The results showed that F4 yielded significant fluorescence values, and we selected F4 as the forward primer. During screening of reverse primers R1 to R5, R3 performed best (**Figure S2B**). Thus, the optimal primer pair was F4+R3, and this pair was used in subsequent experiments.

A PCR test at six gradient annealing temperatures (from 62°C to 67°C, at 1°C intervals) was performed to determine the optimal reaction conditions. PCR products were detected by 2% agarose gel electrophoresis. The results indicated that there was no non-specific amplification at 65°C, and the target band at 187 bp was bright (**Figure S2C**). Therefore, we considered 65°C to be the optimal annealing temperature.

### Specificity of the *N. cyriacigeorgica* CRISPR-PCR Assay

**Figure 2:**
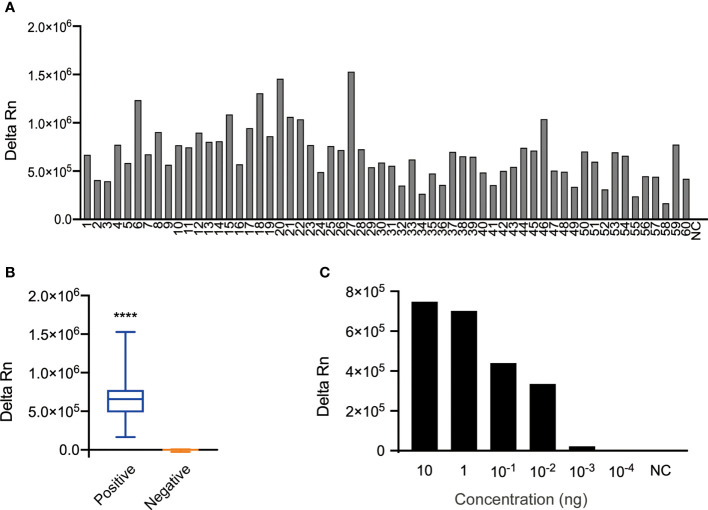
Specificity and sensitivity of the *N. cyriacigeorgica* CRISPR-PCR assay. **(A)** Bar graph showing the fluorescence intensity of the *N. cyriacigeorgica* CRISPR-PCR assay at 37°C for 20 min. 1−60, 60 *N. cyriacigeorgica* strains, including standard strains and clinical strains; NC, negative control. **(B)** Positive detection of *N. cyriacigeorgica* templates (n = 60). Templates of other strains yielded negative results (n = 45). The box-and-whisker plots show fluorescence values generated by the CRISPR-PCR assay at 37°C for 20 min. Unpaired 2-tailed *t-tests* were used to analyse differences from NC (****p < 0.0001). **(C)** Bar graph showing the fluorescence intensities for 10-fold serial dilutions of template detected by the *N. cyriacigeorgica* CRISPR-PCR assay at 37°C for 20 min.

The specificity of the *N. cyriacigeorgica* CRISPR-PCR assay was investigated using 104 N*. cyriacigeorgica* strains and non- *N. cyriacigeorgica* strains (**Table S2**). The results showed that fluorescence signals were detected from 60 N. *cyriacigeorgica* strains at 37°C for 20 min (**Figure 2A**), while other strains (including 19 non-*Nocardia* strains and 25 other *Nocardia* strains) were negative (**Figures S3A, B**). There was a significant difference between positive and negative fluorescence values (**Figure 2B**). Thus, the *N. cyriacigeorgica* CRISPR-PCR assay is highly specific for the detection of *N. cyriacigeorgica*.

### Sensitivity of the *N. cyriacigeorgica* CRISPR-PCR Assay

To evaluate the sensitivity of the *N. cyriacigeorgica* CRISPR-PCR assay, 10-fold serial dilutions of *N. cyriacigeorgica* genomic DNA were used as templates for PCR amplification. The original concentration of DNA template measured by a NanoDrop-1000 instrument was 100 ng/μL. We detected fluorescence values from DNA templates over a concentration range from 10 ng to 10^-4^ ng using a fluorescence reader. The results demonstrated that the LoD of DNA templates with detectable fluorescence was 10^-3^ ng, which was the same with agarose gel analysis (**Figure 2C** and **Figures S4A, B**). And 10^-3^ ng/μl was equivalent to 1.46 × 10^2^ copies/μl, according to DNA copies number formula (6.02 × 10^23^) × (ng/μl × 10^−9^)/(DNA length × 660) = copies/μl.

### Examining the Feasibility of the *N. cyriacigeorgica* CRISPR-PCR Assay for Clinical Samples

**Figure 3:**
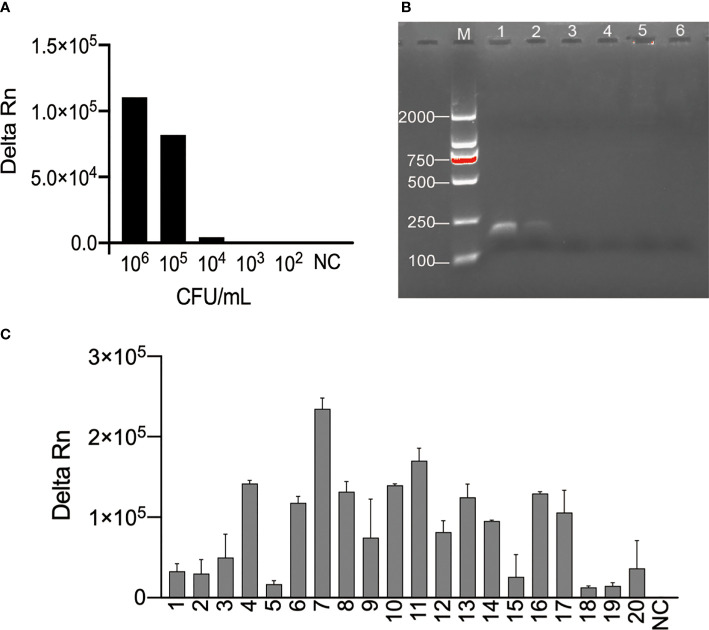
Examination of the feasibility of the *N. cyriacigeorgica* CRISPR-PCR assay. **(A)** Fluorescence signals were measured for a series of *N. cyriacigeorgica* concentrations by *N. cyriacigeorgica* CRISPR-PCR assay at 37°C for 20 min. **(B)** Detection of PCR products by 2% agarose gel electrophoresis. 1−5, concentrations of DNA templates (10^6^, 10^5^, 10^4^, 10^3^, 10^2^ CFU/mL); 6, negative control (NC). **(C)** Fluorescence values for *N. cyriacigeorgica* detection from spiked sputum specimens measured by *N. cyriacigeorgica* CRISPR-PCR assay at 37°C. 1−20, positive samples; NC, negative control.

We explored the sensitivity and specificity of the *N. cyriacigeorgica* CRISPR-PCR assay as a *N. cyriacigeorgica* diagnostic tool for analysis of clinical sputum samples. Negative clinical samples were spiked *N. cyriacigeorgica* to generate a series of final bacterial concentrations (10^6^, 10^5^, 10^4^, 10^3^, 10^2^ CFU/mL). The results showed that the LoD of clinical sputum samples detected by the *N. cyriacigeorgica* CRISPR-PCR assay was 10^4^ CFU/mL (**Figure 3A**). By comparison, the LoD for isolated cultures (**Figure S5**) and agarose gel electrophoresis (**Figure 3B**) was 10^5^ CFU/mL.

Next, we tested DNA templates extracted from 20 *N. cyriacigeorgica*-positive samples and 20 N*. cyriacigeorgica-*negative samples. Using the *N. cyriacigeorgica* CRISPR-PCR assay, fluorescence signals were detected in all 20 spiked sputum samples (**Figure 3C**), while no positive results were obtained from the 20 negative samples (**Figure S6**). These results demonstrated that the *N. cyriacigeorgica* CRISPR-PCR assay can sensitively and specifically detect *N. cyriacigeorgica* in clinical samples.

## Discussion

Currently, the gold standard for the identification of *Nocardia* at the species level is 16S rDNA sequencing, which can achieve detailed species classification and species annotation information. The 16S rDNA sequencing technique relies on pure culture, but cultivation of *N. cyriacigeorgica* requires 2−7 days, which greatly extends the time needed for identification. In addition, 16S rDNA is expensive and difficult to apply in routine laboratories. Matrix-assisted laser desorption/ionisation-time-of-flight mass spectrometry (MALDI-TOF MS) is widely used in clinical practice. However, differences in the cell wall structure of Gram-positive bacteria leads to poor extraction of proteins from microbial cells, resulting in insufficient spectral information for accurate identification (Croxatto et al., 2012). Therefore, it is important to develop a novel method to rapidly and accurately detect *N. cyriacigeorgica*.

The CRISPR-Cas12a system has great potential for nucleic acid detection. Various CRISPR-Cas12a-based detection methods have been reported, including diagnostic techniques for *Vibrio parahaemolyticus* and SARS-CoV-2, with superior specificity and sensitivity compared with other approaches. Isothermal amplification methods are used to detect nucleic acids in combination with CRISPR-Cas techniques. Although the isothermal amplification efficiency is 10−100 times that of PCR, it is easy to form an aerosol and cause false-positive results. Conventional PCR amplification is more stable than isothermal amplification, and has a wider range of clinical applications. Herein, we selected PCR as the nucleic acids amplification method in the first step, which has the advantages of affordability, reagent availability, and stable experimental results. Additionally, adapting conventional PCR to amplify nucleic acids greatly reduces the requirements for dedicated equipment and technical operators. The *N. cyriacigeorgica* CRISPR-PCR assay achieved detection using readily available equipment, which is suitable for routine clinical laboratories.

Compared with quantitative PCR and conventional PCR, the CRISPR-Cas system has the advantage of distinguishing single-base differences. We employed the CRISPR-PCR assay to detect *N. cyriacigeorgica*. At the nucleic acid amplification stage, target sequences were exponentially amplified by PCR primers to ensure high-efficiency nucleic acid detection in the next step. After amplification, CRISPR-Cas12a recognised target sequences *via* crRNA for precise detection with extremely high specificity. The specificity of the *N. cyriacigeorgica* CRISPR-PCR assay was validated using *N. cyriacigeorgica* and other pathogenic strains. The results showed that the assay accurately detected *N. cyriacigeorgica* DNA without false-positive signals from other pathogens, confirming its specificity for *N. cyriacigeorgica*.

There are significant differences in the susceptibility of different *Nocardia* species to antibiotics. The rapid identification of *Nocardia* is helpful for early and rational use of antibiotics. Our CRISPR-PCR assay showed significant advantages compared with traditional detection methods based on growth characteristics, colony morphology, and biochemical reactions, which are time-consuming, laborious, and unable to achieve species-level identification. The CRISPR-PCR assay could directly detect nucleic acids extracted from clinical samples to rapidly and accurately identify *N. cyriacigeorgica*, and the entire process can be completed within 2 h. The LoD of the CRISPR-PCR assay was 10^4^ CFU/mL for clinical sputum specimens, significantly better than 10^5^ CFU/mL for isolated culture. The results showed that CRISPR-PCR enables rapid and sensitive detection of *N. cyriacigeorgica* and avoids the subjectivity of visual observation, hence the assay may be more suitable for the detection of *N. cyriacigeorgica* infection in clinical samples.

Our previous work CRISPR-CPA and CRISPR-PCR were CRISPR-Cas12a-based detection combined with PCR pre-amplification. However, the detection strategies of the two methods were different. In CRISPR-CPA system, a Protospacer adjacent motif site (PAM) sequences TTTA and a protective base were added at the 5’ end of forward primer, and the crRNA was designed at the downstream of the PAM site (Qiu et al., 2021). Nevertheless, CRISPR-CPA may not be suitable to detect *N. cyriacigeorgica*, because the species-specific gene used in this study was not conserved enough, which was due to the high intraspecific variability of *N. cyriacigeorgica*. our CRISPR-PCR assay, TTTA sequence was not used as a recognition site for Cas12a to initiate CRISPR-based nucleic acid detection. We used non-canonical C-containing sequences as PAM sites to guide the Cas12a/crRNA complex to bind to the target sequence. According to previous studies, LbCas12a possessed extensive PAM sites and non-canonical C-containing sequences could be suboptimal PAM sites (Tran et al., 2021; Dronina et al., 2022). The application of Cas12a to nucleic acid detection relies on trans-cleavage activity. The collateral cleavage activity of the CRISPR-Cas12a system is independent of target sequences cleavage and trans-cleavage activity is triggered upon binding of the Cas12a/crRNA complex to target sequences (Chen et al., 2018). Therefore, in this study, our system is sufficient to trigger trans-cleavage activity to detect the target.

The *N. cyriacigeorgica* CRISPR-PCR assay also has several limitations. One limitation of this study is that we did not use *N. cyriacigeorgica* clinical specimens to evaluate our CRISPR-PCR fluorescence detection platform. Clinical samples such as sputum, blood and bronchoalveolar lavage fluid need to be tested in future. Another limitation of this method is that only a single species of *Nocardia* can be identified, and multiple *Nocardia* strains cannot be distinguished at the same time, and this requires further exploration.

## Conclusion

In this study, we established a sensitive and specific detection method that combined the CRISPR-Cas12a system with PCR amplification for detection of *N. cyriacigeorgica* DNA. The intensity of the fluorescence signal can be measured by a fluorescence reader, and the whole process is completed within 2 h. The assay is suitable for applying to conventional microorganism laboratories, and it can reliably detect *N. cyriacigeorgica* using ordinary equipment.

## Data Availability Statement

The original contributions presented in the study are included in the article/**Supplementary Material**. Further inquiries can be directed to the corresponding author.

## Ethics Statement1

The manuscript contains experiments using sputum specimens from human and the study was approved by the Research Ethics Committee of National Institute for Communicable Disease Control and Prevention, Chinese Center for Disease Control and Prevention (No. ICDC-2021004).

## Author Contributions

XL, XQ, and SX conceived and designed the experiments. XL and XQ wrote the manuscript and performed the experiments. YC, LH, YK, YY, SC, and FL analysed the data and contributed reagents. ZL provided financial and administrative support. All authors contributed to the article and approved the submitted version.

## Funding

This work was supported by the Biosafety Key Special Project (2019YFC1200700, 2019YFC1200601) and the National Natural Science Foundation of China (no. 82073624).

## Conflict of Interest

The authors declare that the research was conducted in the absence of any commercial or financial relationships that could be construed as a potential conflict of interest.

## Publisher’s Note

All claims expressed in this article are solely those of the authors and do not necessarily represent those of their affiliated organizations, or those of the publisher, the editors and the reviewers. Any product that may be evaluated in this article, or claim that may be made by its manufacturer, is not guaranteed or endorsed by the publisher.

## References

[B1] Abudayyeh O. O. Gootenberg J. S. Konermann S. Joung J. Slaymaker I. M. Cox D. B.. (2016). C2c2 Is a Single-Component Programmable RNA-Guided RNA-Targeting CRISPR Effector. Science 353 (6299), aaf5573. doi: 10.1126/science.aaf5573 27256883 PMC5127784

[B2] Barnaud G. Deschamps C. Manceron V. Mortier E. Laurent F. Bert F.. (2005). Brain Abscess Caused by *Nocardia Cyriacigeorgica* in a Patient With Human Immunodeficiency Virus Infection. J. Clin. Microbiol. 43 (9), 4895–4897. doi: 10.1128/JCM.43.9.4895-4897.2005 16145170 PMC1234150

[B3] Brown-Elliott B. A. Brown J. M. Conville P. S. Wallace R. J. Jr. (2006). Clinical and Laboratory Features of the *Nocardia* Spp. Based on Current Molecular Taxonomy. Clin. Microbiol. Rev. 19 (2), 259–282. doi: 10.1128/CMR.19.2.259-282.2006 16614249 PMC1471991

[B4] Castro J. G. Espinoza L. (2007). *Nocardia* Species Infections in a Large County Hospital in Miami: 6 Years Experience. J. Infect. 54 (4), 358–361. doi: 10.1016/j.jinf.2006.08.003 16979760

[B5] Chen W. Liu Y. Zhang L. Gu X. Liu G. Shahid M.. (2017). *Nocardia Cyriacigeogica* From Bovine Mastitis Induced *In Vitro* Apoptosis of Bovine Mammary Epithelial Cells *via* Activation of Mitochondrial-Caspase Pathway. Front. Cell Infect. Microbiol. 7, 194. doi: 10.3389/fcimb.2017.00194 28573110 PMC5435817

[B6] Chen J. S. Ma E. Harrington L. B. Da Costa M. Tian X. Palefsky J. M.. (2018). CRISPR-Cas12a Target Binding Unleashes Indiscriminate Single-Stranded DNase Activity. Science. 360 (6387), 436–439. doi: 10.1126/science.aar6245 29449511 PMC6628903

[B7] Condas L. A. Ribeiro M. G. Yazawa K. de Vargas A. P. Salerno T. Giuffrida R.. (2013). Molecular Identification and Antimicrobial Susceptibility of *Nocardia* Spp. Isolated From Bovine Mastitis in Brazil. Vet. Microbiol. 167 (3-4), 708–712. doi: 10.1016/j.vetmic.2013.08.019 24060098

[B8] Conville P. S. Brown-Elliott B. A. Smith T. Zelazny A. M. (2018). The Complexities of Nocardia Taxonomy and Identification. J. Clin. Microbiol. 56 (1), e01419–17. doi: 10.1128/JCM.01419-17 PMC5744224 29118169

[B9] Coussement J. Lebeaux D. van Delden C. Guillot H. Freund R. Marbus S.. (2016). *Nocardia* Infection in Solid Organ Transplant Recipients: A Multicenter European Case-Control Study. Clin. Infect. Dis. 63 (3), 338–345. doi: 10.1093/cid/ciw241 27090987

[B10] Croxatto A. Prod'hom G. Greub G. (2012). Applications of MALDI-TOF Mass Spectrometry in Clinical Diagnostic Microbiology. FEMS Microbiol. Rev. 36 (2), 380–407. doi: 10.1111/j.1574-6976.2011.00298.x 22092265

[B11] Dronina J. Samukaite-Bubniene U. Ramanavicius A. (2022). Towards Application of CRISPR-Cas12a in the Design of Modern Viral DNA Detection Tools (Review). J. Nanobiotechnology 20 (1), 41. doi: 10.1186/s12951-022-01246-7 35062978 PMC8777428

[B12] Gootenberg J. S. Abudayyeh O. O. Lee J. W. Essletzbichler P. Dy A. J. Joung J.. (2017). Nucleic Acid Detection With CRISPR-Cas13a/C2c2. Science 356 (6336), 438–442. doi: 10.1126/science.aam9321 28408723 PMC5526198

[B13] Hamdi A. M. Fida M. Deml S. M. Abu Saleh O. M. Wengenack N. L. (2020). Retrospective Analysis of Antimicrobial Susceptibility Profiles of Nocardia Species From a Tertiary Hospital and Reference Laboratory, 2011 to 2017. Antimicrob. Agents Chemother. 64 (3), e01868–19. doi: 10.1128/AAC.01868-19 PMC7038297 31818815

[B14] Han L. Ji X. Xu S. Fan S. Wang C. Wei K.. (2020). Microbiological Profile of Distinct Virulence of *Nocardia Cyriacigeorgica* Strains *In Vivo* and *In Vitro*. Microb. Pathog. 142, 104042. doi: 10.1016/j.micpath.2020.104042 32045646

[B15] Harrington L. B. Burstein D. Chen J. S. Paez-Espino D. Ma E. Witte I. P.. (2018). Programmed DNA Destruction by Miniature CRISPR-Cas14 Enzymes. Science. 362 (6416), 839–842. doi: 10.1126/science.aav4294 30337455 PMC6659742

[B16] Hashemi-Shahraki A. Heidarieh P. Bostanabad S. Z. Hashemzadeh M. Feizabadi M. M. Schraufnagel D.. (2015). Genetic Diversity and Antimicrobial Susceptibility of *Nocardia* Species Among Patients With Nocardiosis. Sci. Rep. 5, 17862. doi: 10.1038/srep17862 26638771 PMC4671095

[B17] Huang L. Chen X. Xu H. Sun L. Li C. Guo W.. (2019). Clinical Features, Identification, Antimicrobial Resistance Patterns of *Nocardia* Species in China: 2009-2017. Diagn. Microbiol. Infect. Dis. 94 (2), 165–172. doi: 10.1016/j.diagmicrobio.2018.12.007 30679058

[B18] Huang Z. Tian D. Liu Y. Lin Z. Lyon C. J. Lai W.. (2020). Ultra-Sensitive and High-Throughput CRISPR-P Owered COVID-19 Diagnosis. Biosens Bioelectron. 164, 112316. doi: 10.1016/j.bios.2020.112316 32553350 PMC7245202

[B19] Ji X. Zhang X. Sun L. Hou X. Song H. Han L.. (2020). The Heparin-Binding Hemagglutinin of *Nocardia Cyriacigeorgica* GUH-2 Stimulates Inflammatory Cytokine Secretion Through Activation of Nuclear Factor κb and Mitogen-Activated Protein Kinase Pathways *via* TLR4. Front. Cell Infect. Microbiol. 10, 3. doi: 10.3389/fcimb.2020.00003 32117792 PMC7031410

[B20] Kellner M. J. Koob J. G. Gootenberg J. S. Abudayyeh O. O. Zhang F. (2019). SHERLOCK: Nucleic Acid Detection With CRISPR Nucleases. Nat. Protoc. 14 (10), 2986–3012. doi: 10.1038/s41596-019-0210-2 31548639 PMC6956564

[B21] Lebeaux D. Bergeron E. Berthet J. Djadi-Prat J. Mouniée D. Boiron P.. (2019). Antibiotic Susceptibility Testing and Species Identification of *Nocardia* Isolates: A Retrospective Analysis of Data From a French Expert Laboratory, 2010-2015. Clin. Microbiol. Infect. 25 (4), 489–495. doi: 10.1016/j.cmi.2018.06.013 29933049

[B22] Li S. Y. Cheng Q. X. Liu J. K. Nie X. Q. Zhao G. P. Wang J. (2018). CRISPR-Cas12a has Both *Cis-* and *Trans-*Cleavage Activities on Single-Stranded DNA. Cell Res. 28 (4), 491–493. doi: 10.1038/s41422-018-0022-x 29531313 PMC5939048

[B23] Li S. Y. Cheng Q. X. Wang J. M. Li X. Y. Zhang Z. L. Gao S.. (2018). CRISPR-Cas12a-Assisted Nucleic Acid Detection. Cell Discov. 4, 20. doi: 10.1038/s41421-018-0028-z 29707234 PMC5913299

[B24] McTaggart L. R. Richardson S. E. Witkowska M. Zhang S. X. (2010). Phylogeny and Identification of *Nocardia* Species on the Basis of Multilocus Sequence Analysis. J. Clin. Microbiol. 48 (12), 4525–4533. doi: 10.1128/JCM.00883-10 20844218 PMC3008441

[B25] Minero M. V. Marín M. Cercenado E. Rabadán P. M. Bouza E. Muñoz P. (2009). Nocardiosis at the Turn of the Century. Med. (Baltimore) 88 (4), 250–261. doi: 10.1097/MD.0b013e3181afa1c8 19593231

[B26] Qiu X. Xu S. Liu X. Han L. Zhao B. Che Y.. (2021). A CRISPR-Based Nucleic Acid Detection Platform (CRISPR-CPA): Application for Detection of Nocardia Farcinica. J. Appl. Microbiol. 00, 1–9. doi: 10.1111/jam.15424 34936163

[B27] Tran M. H. Park H. Nobles C. L. Karunadharma P. Pan L. Zhong G.. (2021). A More Efficient CRISPR-Cas12a Variant Derived From Lachnospiraceae Bacterium MA2020. Mol. Ther. Nucleic Acids 24, 40–53. doi: 10.1016/j.omtn.2021.02.012 33738137 PMC7940699

[B28] Wang B. Wang R. Wang D. Wu J. Li J. Wang J.. (2019). Cas12aVDet: A CRISPR/Cas12a-Based Platform for Rapid and Visual Nucleic Acid Detection. Anal. Chem. 91 (19), 12156–12161. doi: 10.1021/acs.analchem.9b01526 31460749

[B29] Xiong D. Dai W. Gong J. Li G. Liu N. Wu W.. (2020). Rapid Detection of SARS-CoV-2 With CRISPR-Cas12a. PloS Biol. 18 (12), e3000978. doi: 10.1371/journal.pbio.3000978 33320883 PMC7737895

[B30] Zhang W. S. Pan J. Li F. Zhu M. Xu M. Zhu H.. (2021). Reverse Transcription Recombinase Polymerase Amplification Coupled With CRISPR-Cas12a for Facile and Highly Sensitive Colorimetric SARS-CoV-2 Detection. Anal. Chem. 93 (8), 4126–4133. doi: 10.1021/acs.analchem.1c00013 33570401

