# Supplementary material for: A CRISPR-Cas12a-Assisted Fluorescence Platform for Rapid and Accurate Detection of Nocardia cyriacigeorgica
Source: Front Cell Infect Microbiol. 2022 Mar 2;12:835213. doi: 10.3389/fcimb.2022.835213 (PMC8924655; doi:10.3389/fcimb.2022.835213)
Supplement: Supplementary file 1 [file DataSheet_1.docx]

**Supplementary Materials**

**Table S1.** **Oligonucleotide sequences used in this study**

| Oligonucleotide name | Sequence (5’−3’) |
| --- | --- |
| Forward primer 1 | CCAANNCCGAGATCGTGGTGCC |
| Forward primer 2 | CCCAANNCCGAGATCGTGGTGC |
| Forward primer 3 | CAANNCCGAGATCGTGGTGCC |
| Forward primer 4 | CCGCCCAANNCCGAGATCGT |
| Forward primer 5 | AANNCCGAGATCGTGGTGCC |
| Forward primer 6 | CNNNCCGCCCAANNCCGAGATCGTGGTGCC |
| Forward primer 7 | GNTNCNNNCCGCCCAANNCCGAGA |
| Reverse primer 1 | ACGTGGTTCTCCCATTCGAACC |
| Reverse primer 2 | ACGTGGTTNTNCCATTCGAACC |
| Reverse primer 3 | CGTGGTTCTCCCATTCGAACC |
| Reverse primer 4 | CATTCGAACCGGCCGCCGCAGTTCCAGCT |
| Reverse primer 5 | GTNAGNACGTGGTTNTNCCATTCGAACC |
| crRNA | UAAUUUCUACUAAGUGUAGAU-UGGACCAUGCUGGUGAACAA |
| ssDNA probe | 5’-6-FAM-TTTTTTTT-BHQ1-3’ |

**Table S2. Pathogens tested in this study**

| Strains | Number | *Strains* | Number |
| --- | --- | --- | --- |
| *Nocardia* *cyriacigeorgica* DSM 44484 | 1 | *Nocardia pseudobrasiliensis* | 1 |
| *Nocardia* *cyriacigeorgica* DSM 40350 | 1 | *Nocardia aobensis* | 1 |
| *Nocardia* *cyriacigeorgica* DSM 43004 | 1 | *Nocardia asiatica* | 1 |
| *Nocardia* *cyriacigeorgica* DSM 43005 | 1 | *Nocardia beijingensis* | 1 |
| *Nocardia* *cyriacigeorgica* DSM 43208 | 1 | *Nocardia jinanensis* | 1 |
| *Nocardia* *cyriacigeorgica* DSM 46058 | 1 | *Nocardia Mexicana* | 1 |
| *Nocardia* *cyriacigeorgica* DSM 44730 | 1 | *Nocardia novocastrense* | 1 |
| *Nocardia* *cyriacigeorgica* clinical strains | 53 | *Mycobacterium houstonense* | 1 |
| *Nocardia caishijiensis* | 1 | *Rhodococcus coprophilus* | 1 |
| *Nocardia asteroids* | 1 | *Gordon terrae* | 1 |
| *Nocardia brasiliensis* | 1 | *Tsukamurellaceae* | 1 |
| *Nocardia wallacei* | 1 | *Corynebacterium aurimucosum* | 1 |
| *Nocardia pneumoniae* | 1 | *Corynebacterium imitans* | 1 |
| *Nocardia blacklockiae* | 1 | *Corynebacterium propinquum* | 1 |
| *Nocardia carnea* | 1 | *Streptococcus salivarius* | 1 |
| *Nocardia farcinica* | 1 | *Streptococcus pyogenes* | 1 |
| *Nocardia nova* | 1 | *Streptococcus Pneumoniae* | 1 |
| *Nocardia otitidiscaviarum* | 1 | *Staphylococcus epidermidis* | 1 |
| *Nocardia transvalensis* | 1 | *Staphylococcus succinus* | 1 |
| *Nocardia abscessus* | 1 | *Staphylococcus haemolyticus* | 1 |
| *Nocardia african* | 1 | *Stenotrophomonas maltophilia* | 1 |
| *Nocardia brevicatena* | 1 | *Moraxella catarrhalis* | 1 |
| *Nocardia kruczakiae* | 1 | *Klebsiella pneumoniae* | 1 |
| *Nocardia paucivorans* | 1 | *Escherichia coli* | 1 |
| *Nocardia veterana* | 1 | *Kocuria kristinae* | 1 |
| *Nocardia amikacinitolerans* | 1 | *Salmonella* | 1 |


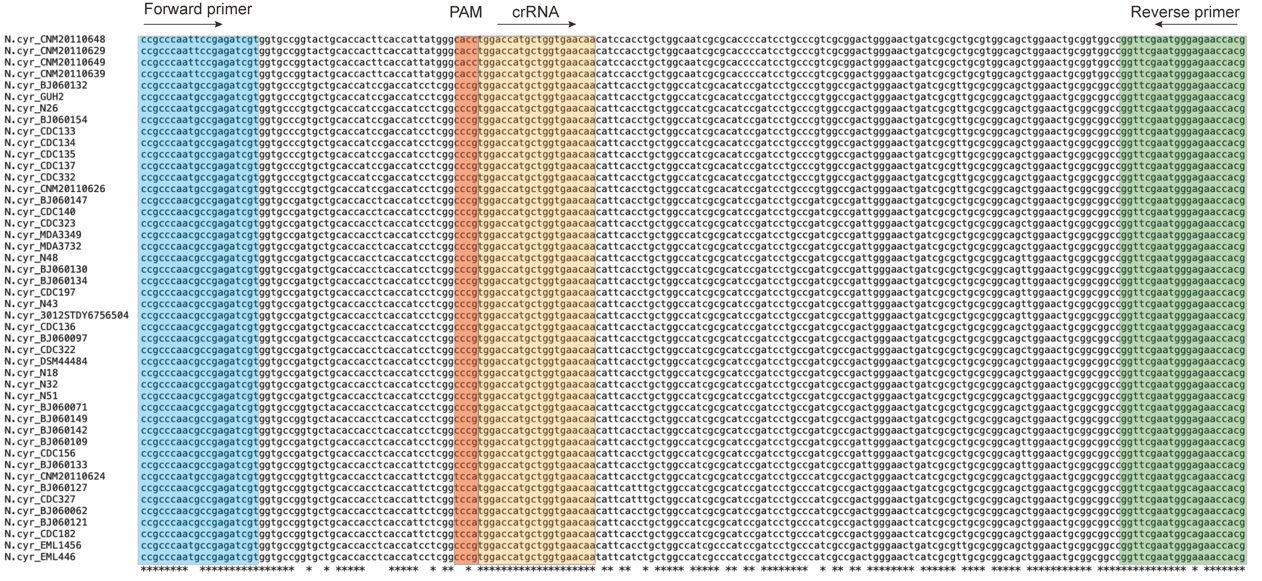


**Figure S1.** Multiple sequence alignment of amplified DNA fragment of *Nocardia cyriacigeorgica*. '*' indicates positions that have a single, fully conserved residue. The positions and directions of forward primer, crRNA and reverse primer was labeled in the figure. Forward primer, crRNA and reverse primer were highlighted in blue, orange and green backgrounds, respectively.


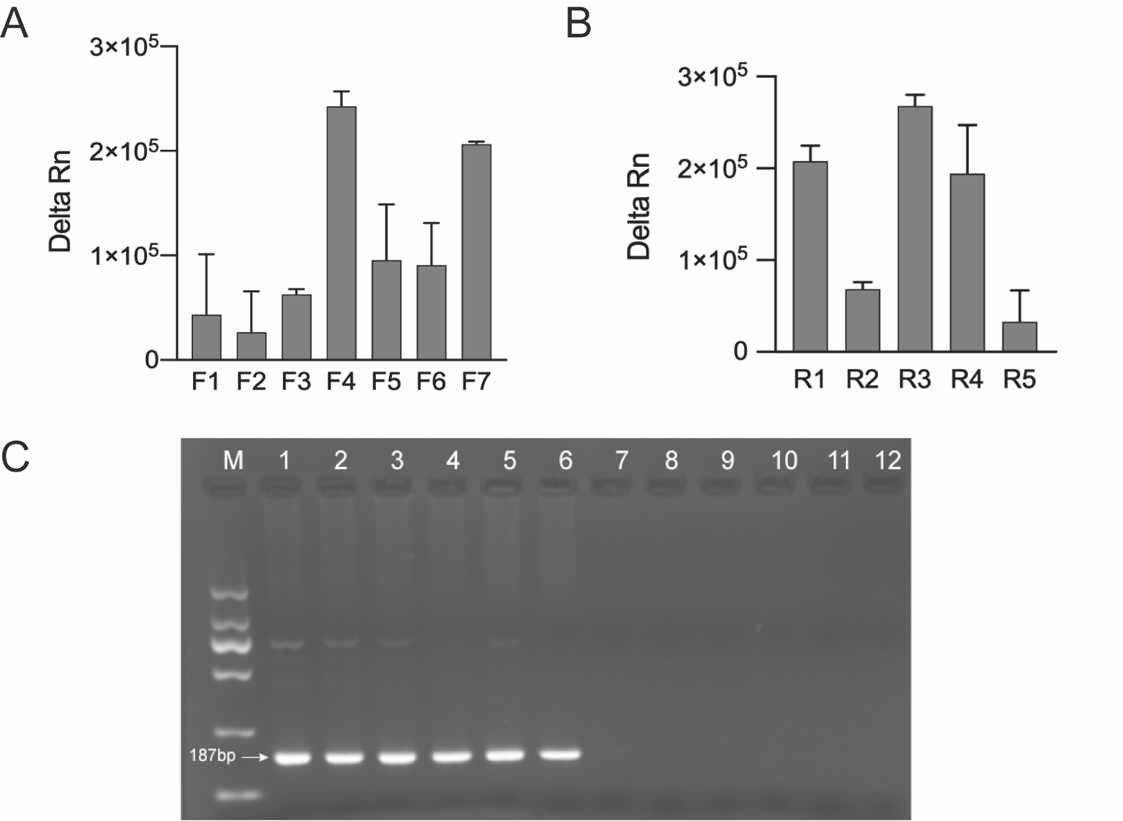


**Figure S2.** Primer screening. (A) Forward primers (F1−F7) were screened against a single reverse primer (R4). (B) Reverse primers (R1−R5) were screened against a single forward primer (F4). (C) Results of 2% agarose gel electrophoresis. 1−6, PCR products with annealing temperatures of 62−67°C；7−12, negative controls at 62−67°C. M, DNA Maker 2000.


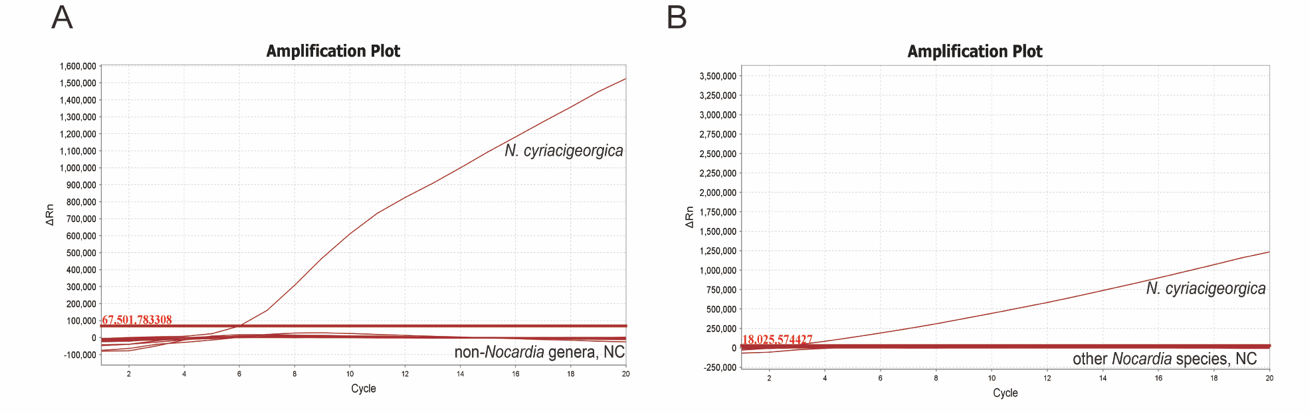


**Figure S3.** Specificity of the *N.* *cyriacigeorgica* CRISPR-PCR assay. (A) Non-*Nocardia* genera produced no fluorescence signal. (B) Other *Nocardia* species produced no fluorescence signal. NC, negative control with no template.


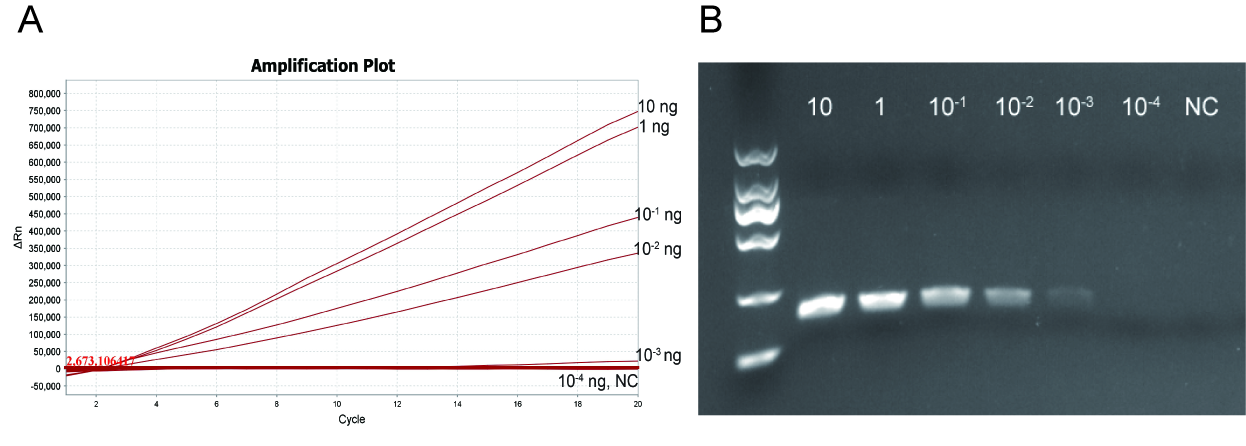


**Figure S4.** Sensitivity of the *N.* *cyriacigeorgica* CRISPR-PCR assay. (A) Fluorescence detection. Fluorescence signals were generated in a real-time PCR instrument at 37°C for 20 min. (B) Agarose gel analysis. Template DNA was serially diluted at 10-fold intervals from 10 ng to 10^-4^ ng. NC, negative control with no template.

**
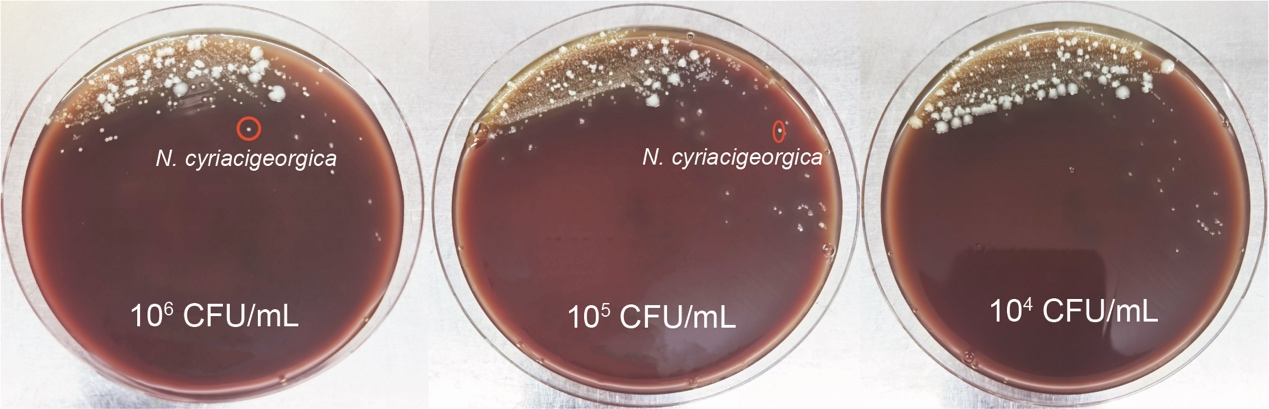
**

**Figure S5.** Blood agar plates with simulated sputum samples containing various concentration *N. cyriacigeorgica*. The plates were incubated at 37°C for 2 days. *N. cyriacigeorgica* colonies were isolated from cultures at 10^6^−10^5^ CFU/mL. No colonies grew from *N. cyriacigeorgica* cultures below 10^5^ CFU/mL.

**
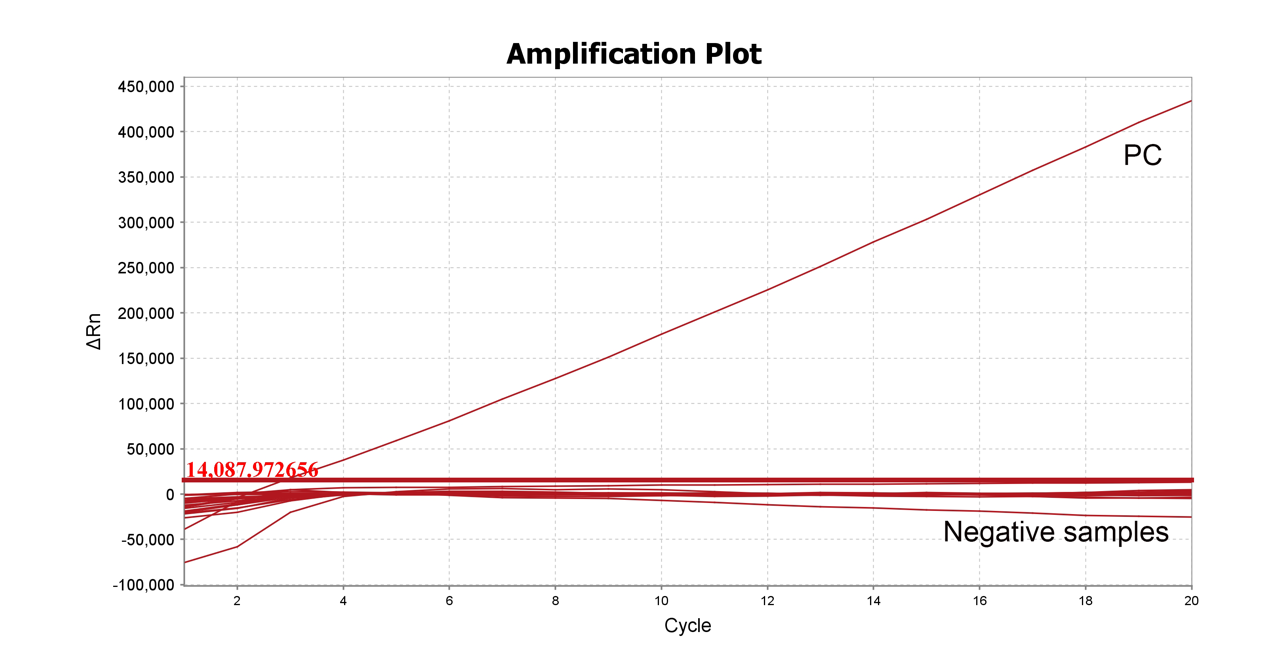
**

**Figure S6.** Feasibility of the *N.* *cyriacigeorgica* CRISPR-PCR assay. Negative samples were 20 clinical samples not spiked with *N. cyriacigeorgica*. PC, positive control.
